# Sexual and Reproductive Healthcare Provided to Women Diagnosed with Serious Mental Illness: Healthcare Professionals’ Perspectives

**DOI:** 10.3390/nursrep15040119

**Published:** 2025-03-28

**Authors:** Glòria Tort-Nasarre, Paola Galbany-Estragués, María Ángeles Saz Roy, Maria Romeu-Labayen

**Affiliations:** 1 Faculty of Nursing and Physiotherapy, University of Lleida, 25198 Lleida, Lleida, Spain; gloria.tort@udl.cat; 2 AFIN Research Group and Outreach Centre, Autonomous University of Barcelona, 08193 Cerdanyola del Vallés, Barcelona, Spain; maria.romeu1@gmail.com; 3 Department of Fundamental and Clinical Nursing, Faculty of Nursing, University of Barcelona, 08907 L’Hospitalet de Llobregat, Barcelona, Spain; 4 Department of Public Health, Mental Health and Mother-Infant Nursing, Faculty of Nursing, University of Barcelona, 08907 L’Hospitalet de Llobregat, Barcelona, Spain; masazroy@ub.edu

**Keywords:** nursing, serious mental illness, professional practice, qualitative research, reproductive health, sexual health

## Abstract

**Background**: Women diagnosed with serious mental illness (SMI) face increased vulnerability and significant risks to their sexual and reproductive health, an issue that is often overlooked in healthcare systems. Aim: This study aimed to explore the sexual and reproductive healthcare provided to women with SMI, based on the perspectives of professionals specialising in mental health and sexual and reproductive health. **Methods**: A descriptive qualitative design was used. Semi-structured interviews were conducted with a purposive sample of professionals from community mental health and sexual and reproductive health in Catalonia (Spain). Data were analysed using thematic analysis. **Results**: Two themes were identified: clinical practice and professional context. The clinical practice theme had three sub-themes: lack of a preventive framework, attention to sexual and reproductive needs, and supporting women in their desire for motherhood and in pregnancy. The professional context theme had four sub-themes: cross-disciplinary coordination, lack of protocols, lack of human resources and time, and lack of training in mental health. **Conclusions**: Mental health professionals and sexual and reproductive health professionals expressed different perspectives about sexual and reproductive healthcare for women with SMI, pointing to a need for greater training and coordination.

## 1. Introduction

Women diagnosed with serious mental illness (SMI) face increased vulnerability and risks to their sexual and reproductive health, including pregnancy, due to a complex combination of biological, social, and environmental factors. They have specific needs that are often not adequately covered, leading to health inequalities [1]. To identify the baseline of the care provided in Catalonia (Spain), we explored the sexual and reproductive healthcare offered to women with SMI, according to professionals in mental health and sexual and reproductive health.

SMI is a group of psychiatric diagnoses that includes bipolar disorder, major depression, and schizophrenia [2] and affects 335 million people worldwide [3]. SMI significantly compromises a person’s ability to function in daily life, generating interruptions in their social, work, and educational spheres. Previous research has documented a range of health issues linked to SMI in women including increased risk of cardiovascular diseases [4], metabolic disorders [5], substance abuse, and higher rates of sexual violence and trauma [6]. Additionally, women with SMI often experience poorer outcomes in managing chronic conditions, lower life expectancy, and significant social and economic barriers to accessing healthcare [7,8]. Women diagnosed with SMI have a higher prevalence of and premature mortality from breast and gynaecologic cancer compared to the general population [4,9,10], and they are less likely to receive breast and cervical cancer screenings [1]. In addition, they experience a reduction in fertility, a side effect of antipsychotics [11]. They also have a higher risk of polycystic ovary syndrome, cancer, and endometriosis than the general population [12]. At the same time, psychiatric diagnoses are associated with a higher probability of unwanted pregnancies [13], which can lead to clinical destabilisation. Likewise, the perinatal period is one of greater fragility, and the presence of psychiatric symptoms (depressive, manic, psychotic) during pregnancy makes women with SMI especially vulnerable [14]. Having a diagnosis of SMI increases the risk of adverse pregnancy outcomes and increased neonatal morbidity [15]. For women with SMI, it can be difficult to care for a child while also managing their illness [16]. Finally, socioeconomic inequalities that often accompany mental illness can further exacerbate the difficulties women with SMI face in accessing and receiving adequate reproductive health services. Tailored programmes that focus on improving well-being and reproductive rights for women with mental health challenges are essential in addressing the specific needs of this vulnerable group. In some countries, women with SMI are provided with medications to prevent unwanted pregnancies, ensuring that they have access to family planning options [17,18].

Despite the prevalence and severity of sexual and reproductive health conditions in women with SMI, the lack of focus on sexual and reproductive health as an integral part of healthcare is evidenced by the frequent underestimation of sexual and reproductive needs by generalist and specialist health professionals [11]. This mismatch has serious and predictable repercussions, such as not obtaining informed consent before initiating medication that can affect foetal development, harmful psychiatric outcomes due to abrupt discontinuation of psychotropic medication during pregnancy, inadequate postpartum and peripartum follow-up of high-risk patients, and limited attention to psychosocial needs [19,20].

There is a lack of research on the perspectives of professionals who work in direct care with women diagnosed with SMI on the sexual and reproductive healthcare that such women receive. It is important to address this gap because the perspectives of frontline care professionals are essential for enhancing the quality and accessibility of sexual and reproductive healthcare for women with SMI. Nurses work in health promotion and disease prevention; for this reason, research by nurses is key to incorporating knowledge that can improve aspects that are still lacking in sexual and reproductive healthcare for women living with SMI, to enhance quality of life and longevity. A first step is to identify the baseline of the care provided, so that improvements can be made from this starting point. Therefore, we defined the following research question: How do professionals specialising in mental health and sexual and reproductive health describe the sexual and reproductive healthcare provided to women diagnosed with SMI? Incorporating these perspectives can help ensure that their specific needs are better recognised and met.

## 2. Materials and Methods

### 2.1. Study Design

A qualitative descriptive study [21,22] was conducted to collect information about the perspectives of the health professionals in mental health and sexual and reproductive health in Catalonia (Spain) who work with women diagnosed and living with SMI. This design is well-suited to gaining a deeper understanding of practice in applied disciplines and is especially relevant when the goal is to understand participants’ perspectives and experiences [22].

### 2.2. Context and Participants

Spain offers free, public healthcare, which is managed by each regional government [23]. Additionally, private care is available through direct services and insurance plans. While private care tends to have shorter waitlists, the two systems are comparable overall, and professionals in both systems follow the same general protocols. Our research took place primarily in the public health network of the region of Catalonia between March and September 2023. Considering strategic and pragmatic criteria, we chose participants who could offer a rich cross section of data—psychiatrists, mental health nurses, gynaecologists, and midwives—through purposive sampling [24]. The inclusion criteria were (a) being a health professional working in the province of Barcelona, (b) working in either mental health or sexual and reproductive health, (c) being a provider for women diagnosed with SMI, (d) having a minimum experience of two years, and (e) being willing to participate. There were no exclusion criteria. Participants were recruited by one of the researchers who had contact with mental health and primary care service management teams. These teams were asked to identify sexual and reproductive health professionals whose perspectives could be valuable to the study and who were interested in speaking with us. One author contacted participants by telephone and invited them to participate in the study. Of the ten professionals that were invited to participate, two gynaecologists declined because they had not treated women with SMI, leaving an initial sample of eight professionals, each from a different Catalonian health centre.

Four participants were mental health professionals and four were sexual and reproductive professionals. The four participants in the mental health area, two psychiatrists and two mental health nurses, came from public community mental health centres, which are part of the public health network and integrated into the primary care system. These centres offer diagnosis, treatment, and follow-up services for people with mental illnesses. Four participants were specialists in sexual and reproductive health. One gynaecologist worked at a private assisted reproduction centre. The other three participants were midwives or gynaecologists from different Sexual and Reproductive Health Care Centres (ASSIR in Catalan), also part of the public network and the primary care system. In these centres, midwives provide care during pregnancy, childbirth, and the postpartum period, as well as promoting sexual and reproductive health. Gynaecologists, in contrast, offer specialised services, including gynaecological check-ups, pregnancy monitoring, and treatment of reproductive health conditions (Table 1).

### 2.3. Data Collection

Following the principles of descriptive qualitative study design, we collected data using semi-structured interviews [24] between March and September 2023. One of the authors interviewed the mental health professionals and another one interviewed the sexual and reproductive health professionals. The researchers had no prior contact with the participants. Interviews were conducted by videoconference and lasted 45–60 min. The interviews were performed in Catalan or Spanish, depending on the preference of the participant. All interviews were recorded and transcribed with the participants’ written consent. We used a semi-structured interview guide, which we adjusted slightly after the first interview. The guiding questions were as follows:

1. In your opinion, how is sexual and reproductive healthcare provided for people with SMI?
2. What experience do you have in counselling women with SMI on sexual and reproductive health, prevention, pregnancy, and motherhood?
3. From your experience, how often do women with SMI consult on these issues and how are these consultations handled in terms of specific care plans and protocols?
4. From your point of view, have women with SMI required a different approach and how you have handled these situations?

Notes were taken during the interviews to help guide the questions. We defined saturation as the point at which no new information was identified from interview responses [25]. We reached saturation by the sixth interview and proceeded to conduct two final interviews to confirm saturation, at which point no further participants were recruited.

### 2.4. Data Analysis

The interviews were transcribed and analysed in the original Catalan or Spanish. Interview notes were not included in the analysis. Operating within our descriptive qualitative design, we followed the five-stage methodological framework proposed by Braun and Clarke to conduct a thematic analysis [26], with an inductive approach based on the experience of the participants, without imposing a prior theoretical framework and with the support of the qualitative analysis software package Dedoose Version 7.0.23. (www.dedoose.com). In the familiarisation stage, the researchers immersed themselves in the data by reading and re-reading the transcripts to gain a deep understanding of it, noting initial thoughts and reflections. During code generation, the researchers identified and marked relevant segments, which were grouped into categories. In the search for themes, they sorted these codes into potential themes and examined their relationships. They then reviewed and refined the themes, ensuring clarity and accuracy. Finally, they defined and named each theme, explaining its contribution to answering the research question.

### 2.5. Trustworthiness

This study meets the criteria of credibility, transferability, dependability, and confirmability, which ensure trustworthiness in qualitative research [27]. Credibility was ensured because the results reflect the reality studied. We conducted a literature search for points of comparison and contrast, and we consulted experts in qualitative methodology and triangulated the analysis across the researchers. Transferability was achieved by providing a description of the healthcare context and the sample. Regarding dependability, we carried out constant review and a detailed description of how we collected, analysed, and interpreted the data. Confirmability was ensured by constant reflection that encouraged us to consider our own positionality.

### 2.6. Research Team and Reflexivity

The research team was made up of nurses that hold doctorates and have experience in qualitative research. Reflexivity is important in ensuring that researchers reach an accurate interpretation of the data. In this sense, team members have been aware of the necessity of maintaining distance with their own nursing practice, which has contributed to identifying the influence of their own nursing practice during the analysis process. The fact that none of us currently work in patient care helped reduce the risk that our own experiences and opinions would introduce bias into study outcomes. The research team resolved disagreements by consensus. The Consolidated Criteria for Reporting Qualitative Research (COREQ) were used [28].

### 2.7. Ethical Considerations

This study was approved by the Universidad Autónoma de Barcelona (Autonomous University of Barcelona) Ethics Committee for Animal and Human Research with the reference code CA36 and approval date 21 February 2022. Participants were provided with oral and written information explaining that their participation was voluntary and that they could withdraw from the study at any time. All participants signed an informed consent document, which explained that interviews would be audio-recorded. Interviews were anonymised by replacing names with an alphanumeric code in adherence with Spain’s Organic Law 3/2018 on Personal Data Protection. This study forms part of a multi-disciplinary joint project REPROMOB: Repro-flows in Europe, North Africa, and Latin America: peoples and gametes mobilities in the fragmented context of trans-national regulation of assisted reproduction and adoption. We report results from the nursing portion of the study.

## 3. Findings

Eight professionals participated in the study. Table 1 shows their main characteristics. Through the thematic analysis, we identified two themes, subdivided into seven sub-themes (Table 2).

### 3.1. Clinical Practice

A lack of a preventive framework for sexual and reproductive health was identified by the mental health professionals, which are the group that reported the greatest continuity of care for women diagnosed with SMI. Specifically, participants in mental health reported that no preventive actions are conducted at their centre to reduce the risk of STIs and unintended pregnancies among this population.

*The truth is that we do nothing about prevention in terms of what would be sexually transmitted diseases. You focus on medications and don’t usually ask much more about sexual issues.*

P1. Psychiatrist.

Midwives reported that women with an SMI may not be screened properly unless a targeted approach is performed.

*It’s been seen that these women tend to seek less advice when it comes to things like family planning, pap smears, and all that. Maybe what’s needed is a more targeted approach for these women, encouraging things like gynaecological check-ups and, especially, regular breast self-exams.*

P8. Midwife.

At the same time, we observed that care for sexual and reproductive needs is approached differently depending on the specialty. Psychiatrists found exploring the sexual and reproductive sphere particularly challenging.

*It’s a topic you don’t address directly because I think it can be tough due to their potential psychopathology, like when they’re experiencing paranoid moments. So, it can be a sensitive and invasive topic, and it needs to be handled smoothly and based on the personal context, so to speak.*

P2. Psychiatrist.

Therefore, they focus attention on the side effects of psychiatric medication, as mentioned above by P1.

In contrast, mental health nurses described a more focused approach to providing information and generating a climate of trust between the woman and the professional.

*I try not to be too invasive during examinations, aiming to make the visits more than just ticking off a checklist. Instead, I focus on creating an environment where the person feels comfortable enough to talk about what really matters to them.*

P3. Mental health nurse.

Participants highlighted the importance of the person’s clinical stability in addressing their needs.

*If someone with a serious mental illness is stable and comes in for a pap smear, a contraception issue, or an STI problem, you handle it the same way—you don’t treat them any differently*.

P8. Midwife.

It should be noted that SMI does not prevent professionals from carrying out standard technical procedures in the same way as for women without an SMI.

*The process doesn’t change whether a person has a prior mental health diagnosis or not. They still need to go through the same steps, like having ultrasound checks, getting hormone injections, and whatever else is needed. Insemination, in vitro… In other words, this is standard for everyone.*

P5. Gynaecologist.

Psychiatrists and gynaecologists agreed that stabilisation of the SMI is crucial for supporting women in their desire for motherhood and in pregnancy, as well as their ability to care for their children.

*What I would focus on isn’t just the diagnosis, but the person beyond it—how they view their own life and what stance they take regarding their situation.*

P1. Psychiatrist.

*It’s also important to talk with a psychiatrist to adjust medication doses and find the lowest effective doses that are the least likely to be teratogenic. Once she sees that she’s stable on her current medication and is feeling well, she can proceed with the pregnancy.*

P6. Gynaecologist.

The assessment of economic factors, along with family and social support, is essential, and professionals such as midwives and gynaecologists play a crucial role in providing guidance and support in these areas.

*If her condition is under control, she wants to be a mother, feels capable, and has the social and family support to do it, then, I don’t see why not. At the end of the day, everyone is free to do what they want, right?*

P5. Gynaecologist.

Supporting women in their desire to be mothers sometimes means addressing frustrations over the inability to conceive or to care for their children. Validating fears and providing empathetic listening emerge as crucial tools in supporting women with an SMI.

*It’s an approach rooted in the frustration of not being able to do things normally. So, in the end, what you address is that: listening, validating the loss, and working through the grief, if that’s the case.*

P2. Psychiatrist.

There are women whose ability to have children has been questioned by mental health teams.

*What often happens, and I’ve seen this, is that when you talk to women of childbearing age, they already come with a learned narrative because they’ve been told, often during a hospital stay or similar situation, “You won’t be able to have children”.*

P6. Gynaecologist.

Likewise, understanding the vulnerability of women with an SMI during pregnancy requires sensitive and compassionate care that balances individual aspirations with clinical and social reality.

*So, since they are already more vulnerable and, additionally, many may experience crises related to their underlying condition in the postpartum period, a lot of them come in saying, “Wow, will I be able to handle this? Will I stay stable? Will I have enough support from my family to manage everything that’s coming my way?”*

P7. Midwife.

Concerns about the future of child-rearing are also part of reproductive care, as reported by sexual and reproductive care specialists.

*We see every day that there is a lot of fear… Women express a deep concern that they might be separated from their children because they believe they can’t care for them properly. And with a condition like this, I think it’s very important because it adds an extra layer of concern.*

P8. Midwife.

*Many midwives also offer childbirth preparation classes, which can provide them with a support network, a bit more stability, and the chance to meet others who are going through similar life stages. We also try to connect them with the midwife and help them build a support network. And with the paediatricians.*

P6. Gynaecologist.

### 3.2. Professional Context

With respect to the professional context, key differences were observed between the contexts of the two specialties. Specifically, mental health specialists identified more barriers to care compared to sexual and reproductive healthcare professionals, who described more facilitating factors.

For example, sexual and reproductive health specialists described good cross-disciplinary coordination with mental health as a strong point of ongoing care for these women.

*When community mental health services alert us that a woman has been identified with an STI, hasn’t kept up with her pap smears, or has never had a mammogram, these women have a direct pathway; we schedule them as quickly as possible, ideally within the same week, because we need to address their needs urgently.*

P8. Midwife.

*We also make sure to contact the psychiatrist, because sometimes during the pregnancy, her condition can worsen, and hospitalisation may be necessary. So, we always maintain a two-way communication, both with psychiatrists contacting us and us contacting them. We also stress the importance of working closely with the midwife. She plays a key role in offering support that’s less about the medical side and more about helping with fears and providing emotional support.*

P6. Gynaecologist.

However, mental health professionals reported that the degree of coordination depended on the centre.

*There’s usually no regular coordination or knowledge between teams about which cases are being handled together. Unless it’s a very specific case that requires direct contact, we don’t do that.*

P3. Mental health nurse.

The lack of standardised protocols is observed as an added difficulty, on the part of mental health, compared to the existing protocols in sexual and reproductive health.

*There is no protocol as such for now. So, I think everyone just does the best they can.*

P1. Psychiatrist.

*There is no protocol, there’s not one… No, we don’t have any specific protocol.*

P4. Mental health nurse.

Another of the issues described was a lack of human resources and time, which influences the quality of the care provided.

*In a 20 min visit every three or four months, well, you tell me. We’d need more time and more staff. We would need to have a proper amount of time for visits and enough professionals, you know, with decent ratios, to be able to tackle these issues.*

P2. Psychiatrist.

This lack of time could be a barrier to building a professional relationship, which adds challenges to supporting women with an SMI in reproduction, pregnancy, or parenting from a mental health perspective.

*There’s no prevention for anything because there’s no time to do anything. You’re constantly putting out fires, and with just one visit every three months, are you really going to start talking about delicate, sensitive stuff, right? Personal and meaningful things. I mean, of course, you need to have a proper connection, and you need to have some continuity with the visits.*

P2. Psychiatrist.

Also, the lack of mental health training among sexual and reproductive health professionals was identified as an area for improvement

*I think there also needs to be training and education for professionals in other fields when it comes to dealing with mental health patients. Because there’s a lot of ignorance about it. In fact, a lot of people get scared just at the sight of a psychiatric patient*.

P2. Psychiatrist.

*I think there should be training provided to help address these women’s needs and, let’s say, normalise their condition. A lot of times we really need more tools to better understand their condition, how to approach it, and how to connect with these women.*

P7. Midwife.

## 4. Discussion

Our study adds to the literature by presenting the perspective of professionals from two specialties that provide care to women diagnosed with SMI: mental health and sexual and reproductive health. Our analysis reveals clear similarities and differences in the views of these two sets of professionals regarding clinical practice and professional context in the management of the sexual and reproductive health of women with SMI.

With respect to clinical practice, participants pointed to a lack of a preventive framework, which would be key for reducing the risk of STIs and detecting health conditions. Sexual and reproductive health professionals have preventive interventions for the general population, like screenings for early detection of breast or cervical cancer which are essential to improving overall health outcomes and reducing the burden of preventable diseases [29]. Hughes et al. [30] reported that only 54% of people with SMI had ever accessed sexual health services.

Our participants recognised that women diagnosed with SMI might not be adequately screened for these conditions. While sexual and reproductive health teams aim to provide the same level of care to women with SMI as to the general population, they acknowledged that this group is more vulnerable and may face additional barriers to receiving the necessary screenings and care. According to Natividad et al. [31], mental health teams have shown that preventive actions do not include interventions for sexual or reproductive health issues because they are not considered a priority. This situation is compounded by the fact that women diagnosed with SMI rarely seek care for their sexual and reproductive health. Generally, women diagnosed with SMI and cancer may pay less attention to their overall physical health and seldom ask for help [32]. Additionally, they feel uncomfortable discussing sexual and reproductive health topics with professionals [33].

Our study emphasises the perspectives of professionals on prevention. On one hand, sexual and reproductive health teams recognise that women with SMI are a vulnerable group with greater difficulties in accessing screenings, but they lack procedures to address these barriers. On the other hand, it is notable that mental health professionals report significant difficulties in addressing these issues. Our findings highlight that unless women with SMI explicitly request it, mental health professions do not address sexual and reproductive health concerns. This is because these topics might be sensitive for the woman, and they prioritise trust and the therapeutic relationship over addressing sexual and reproductive healthcare. This approach, while well-intentioned, may create a gap in care as it reduces the likelihood of addressing important issues such as pregnancy, parenting, gynaecological screenings, prevention of STIs, and family planning.

Given the finding that mental health professionals are committed to maintaining the therapeutic relationship above all else, more research should be conducted to understand which specialist teams and which professionals should carry out prevention actions related to sexual and reproductive health in women diagnosed with SMI. It is also important to consider that mental health teams are interdisciplinary and incorporate various professional perspectives. According to our findings, while psychiatrists focus on pharmacological treatment and clinical stability, mental health nurses concentrate on creating a climate of trust and responding to women’s information needs, which may also include questions about physical health; as highlighted by Martínez et al. [20], this involves coordinating with other specialties to prevent gaps in comprehensive care and to achieve objectives related to the prevention of sexual and reproductive health issues and overall health.

Regarding supporting women in their desire for motherhood and in pregnancy, participants agreed that clinical stability is a fundamental prerequisite for women diagnosed with SMIs to achieve the autonomy necessary for embarking on pregnancy and parenthood. Previous studies show that this population often encounters resistance from health professionals when they want to become mothers [34,35].

Our study indicates that it is essential to address issues related to frustrations and losses associated with the inability to conceive or adequately care for children, as well as to validate fears and actively listen to vulnerabilities. The psychiatrists in our study saw this as crucial for promoting more humane and effective care. Another contribution of this study is to identify the perspective of mental health nurses regarding care related to the desire for motherhood and pregnancy among women diagnosed with SMI. Nurses emphasised the need to focus not only on maintaining stability and autonomy but also on adopting a broader perspective that includes caring for the family environment, the social context, and the economic situation.

The professional context theme has revealed differences between the experiences and perspectives of specialists in mental health and sexual and reproductive health. While sexual and reproductive health professionals described positive factors, such as cross-disciplinary coordination with mental health professionals or other medical specialties, their mental health counterparts identified more barriers to effective collaboration. In this sense, an important difference stands out: participants from sexual and reproductive health (who worked at four different centres) reported having quick and easy communication with mental health teams when they needed to consult for a woman who had an SMI diagnosis. In contrast, the mental health professionals (also from four different centres) pointed out the lack of strategies to be able to provide adequate care for prevention in sexual and reproductive healthcare. The difference in opinions is related to the lack of general guidelines regarding the coordination between different services and specialists. The participants came from eight different services and health institutions, each with its own model of coordination between services and specialties. In some cases, this coordination relied more on individual decisions than on established coordination protocols.

Beyond individual professional decisions, it is essential to establish standardised protocols and team and institutional strategies to align different professional practises and enhance the protection of women with SMI. Previous studies propose solutions for coordinating oncological treatments in women with mental health diagnoses, such as standardised protocols to provide clear tools for professionals [11,33]. Our results bring together the perspectives of psychiatrists, gynaecologists, mental health nurses, and midwives—specialists who provide care to women diagnosed with SMI.

Addressing systemic shortcomings requires a re-prioritisation of clinical approaches, with promising strategies including integrated care models and improved training in sexual and reproductive health for health professionals [36]. In line with these findings, our participants perceived a lack of response within the Catalonian healthcare system to the specific needs of women with SMI. It is the responsibility of institutions to address this gap and enhance sexual and reproductive health outcomes for this group.

Lack of human resources and time during consultations are adverse factors for preventive activities related to sexual and reproductive health, as well as pregnancy and parenting. This is because professionals must prioritise addressing immediate demands, aligning with previous findings [32,37,38]. Our findings provide perspectives from the two specialties regarding this topic. Sexual and reproductive health teams can provide quick preventive recommendations in the same way they do for women without an SMI diagnosis during the same visit. However, mental health teams consistently point out that the limited consultation time does not allow them to address topics beyond clinical care, treatment, and family and social situations.

The lack of mental health training of teams from other specialties has been identified as an adverse factor in other studies [37,38]. According to our findings, training and integration between mental health services and sexual and reproductive health services are highly necessary. Training should encompass the essential knowledge that mental health professionals need about sexual and reproductive healthcare programmes. Likewise, sexual and reproductive health professionals need a deeper understanding of the needs of women with an SMI diagnosis.

## 5. Strengths and Limitations

This study has some limitations. First, the sample of this study was small, and therefore care is warranted in extrapolating the findings to other settings in Catalonia or beyond. The diversity in the background of the professionals introduces significant contextual variation, as differences in organisation, available resources, and clinical approaches can influence the practises and opinions expressed. Therefore, it is crucial to consider these differences when interpreting the findings, which are not meant to be generalisable but rather to provide a snapshot of the experiences and observations of these health professionals, which can serve as a basis for further research.

Another limitation is the potential for social desirability bias, which could have led participants to provide agreeable responses. One way we tried to mitigate this bias was by using an interviewer external to the participants’ healthcare team, with the hope that the participant would feel freer to express their true opinions when speaking to someone outside their immediate work environment.

## 6. Recommendations for Further Research

Future studies should explore these initial findings with a larger sample both within the studied centres and across a greater number of centres. Another future direction could focus on conducting comparative studies to assess whether the differences observed between mental health and reproductive health in Catalonia are also present in other regions of Spain and in other countries. This would help identify common patterns or divergences, thereby enhancing policies and services in both areas of care.

## 7. Implications for Policy and Practice

This study provides insight into the factors that influence the healthcare of women with SMI according to the perspective of mental health and sexual and reproductive health professionals in primary care. These findings can serve as a basis for improving the effectiveness of care for this vulnerable group. It is essential to optimise programmes for the prevention and detection of STIs and gynaecological and breast cancer in women with SMI, since these women require specialised care that, according to participants, is not offered in our health context. Such care would ensure the early detection and adequate treatment of diseases that often go unnoticed due to their mental state.

The creation of “bridge” teams and the implementation of additional resources for comprehensive support during pregnancy, childbirth, and the postpartum period are recommended. These teams could facilitate coordination between services and ensure continuous care tailored to the specific needs of women with SMI, thus overcoming barriers in care. In addition, it would be appropriate to involve women with SMI in the design and evaluation of programmes or protocols, which would ensure that services are more relevant and tailored to their needs, thus promoting patient-centred care. It is also essential that health professionals strengthen their mental health training, which would improve understanding of these women’s experiences and allow their sexual and reproductive needs to be addressed more effectively.

## 8. Conclusions

This study presents the perspectives of health professionals from both mental health and sexual and reproductive health teams regarding the care of women diagnosed with SMI. Their insights provide a deeper understanding of the current approaches to sexual and reproductive healthcare for these women. While sexual and reproductive health professionals recognised positive factors such as cross-disciplinary coordination with mental health professionals, mental health teams identified more barriers to effective collaboration. The lack of a preventive framework and standardised protocols hinders effective care, emphasising the need for institutional strategies to align professional practises. Additionally, a shortage of human resources and time during consultations, as well as inadequate mental health training among professionals from other specialties, were identified as key challenges for providing comprehensive care. These factors must be addressed to improve sexual and reproductive health services for women with SMI.

## Figures and Tables

**Table 1:** Sample profile and participant characteristics.

Participant Code	Profession	Type of Centre	Sex	Age	Years of Experiences
P1	Psychiatrist	Specialised primary care	Female	37	9
P2	Psychiatrist	Specialised primary care	Male	46	17
P3	Mental health nurse	Specialised primary care	Male	46	23
P4	Mental health nurse	Specialised primary care	Female	44	22
P5	Gynaecologist	Hospital reproductive health unit	Female	33	3
P6	Gynaecologist	Specialised primary care	Female	44	13
P7	Midwife	Specialised primary care	Female	44	16
P8	Midwife	Specialised primary care	Female	42	18

**Table 2:** Themes, definitions of themes, sub-themes, and definitions of sub-themes.

Themes	Definitions of Themes	Sub-Themes	Definitions of Sub-Themes
Clinical Practice	Professionals’ perceptions of the sexual and reproductive healthcare received by women diagnosed with SMI in their daily clinical practice	1. Lack of a preventive framework	1. A lack of specific preventive objectives for the sexual and reproductive healthcare of women diagnosed with SMI, among both mental health teams and sexual and reproductive health teams
		2. Care for sexual and reproductive needs	2. The two kinds of specialists have different views of the sexual and reproductive health needs of women diagnosed with SMI
		3. Supporting women in the desire for motherhood and in pregnancy	3. Perception of professionals regarding the challenges faced by women diagnosed with SMI in deciding whether to have children and during pregnancy and early motherhood
Professional context	Differences between the specialisations regarding views of the sexual and reproductive healthcare received by women diagnosed with SMI	4. Cross-disciplinary coordination	4. The two kinds of specialists have different views of the continuity of care across disciplines
		5. Lack of protocols	5. Lack of coordination between different protocols
		6. Lack of material resources and time	6. Lack of resources of mental health teams to conduct preventive actions
		7. Lack of mental health training	7. Need for sexual and reproductive health teams to improve their knowledge of mental health

## Data Availability

The data presented in this study are available on request from the corresponding author.

## Public Involvement Statement

There was no public involvement in any aspect of this research.

## Guidelines and Standards Statement

This manuscript was drafted against the Consolidated Criteria for Reporting Qualitative Research (COREQ).
